# Book Review: East Asian Perspectives on Silence in English Language Education

**DOI:** 10.3389/fpsyg.2020.631209

**Published:** 2021-01-12

**Authors:** Ping Wang, Honggang Liu

**Affiliations:** School of Foreign Languages, Northeast Normal University, Changchun, China

**Keywords:** East Asian perspectives, silence, contextual factors, theoretical approaches, teaching practice

Silence in foreign language learning has attracted much attention in the field of language education since the 1990s. Previous mainstream studies indicated that silence is a negative and impeditive learning behavior to avoid interaction, which is not conducive to language learning (p. 6). However, according to the contributors of this volume, silence may not necessarily be a hindrance to learners and may play a facilitating role in language teaching. This edited book mirrors this finding by presenting several empirical studies of silence in language learning in the context of East Asia and provides insightful theoretical and practical analysis with far-reaching implications for future studies.

The volume consists of nine chapters, which are grouped into four parts. Chapter 1 provides an overview of the studies under discussion and the main goals of the collection. Chapters 4, 5, and 8, the second part of the book, focus on the relationship between silence and learners' psychological factors, such as anxiety (Chapters 4 and 5) and willingness to communicate (WTC) (Chapter 8). Chapters 2, 3, 6, and 7—as the third part of the book—explore the relationship between silence and contextual factors, involving the influences of task type (Chapter 2), teacher talk (Chapter 3), teaching paradigm (Chapter 6), and classroom activity type (Chapter 7) on silence. Chapter 9, a summary of the collection, focuses on using the methods of action research and case study to investigate the silence from the perspective of dynamic system theory (DST).

This volume stands out because of its contributions toward demonstrating diverse theoretical perspectives in viewing silence, demystifying the roles of contextual factors in constructing silence, and offering practical insights into teaching practice. As such, it is a highly recommended reading for researchers and teachers alike.

First, this volume presents a variety of theoretical views on the study of silence, reflecting a trend of the social turn (Block, 2003) and the complex system turn (Larsen-Freeman, 1997) in the second language acquisition and language learners' psychology. This edited book explores the learners' classroom silence behavior from the cognitive, sociocultural, interactional, and complex theoretical perspectives, and conceptualizes silence as a complex, multidimensional, and highly contextual phenomenon. For example, Peng (Chapter 8) explores the complicated dynamic relationship between L2 WTC and silence from the perspective of dynamic system theory. It is found that WTC itself can be conceptualized as explicit intention to communicate and as implicit WTC sometimes, that is, silent WTC. In this study, five different states of interaction between the two are formed by the interplay of internal and external contextual factors.

Second, contextual factors are foregrounded in this volume, for they play vital roles in constructing and interpreting silence in language learning. Silence is formed in different levels of ecological systems, where various social and cultural values are situated from micro to macro levels. In prior studies, it is believed that the influence of Confucian cultural values on East Asian learners may lead to silence in class due to modesty or shyness (Hu, 2002). In the micro classroom environment, teachers often actively create a context to stimulate students' participation. Those who do not participate in it are labeled as inactive/passive learners. However, this volume gets a fresh insight into the relationship between environment and silence. The standard to evaluate whether a classroom environment is ideal should not depend on the learners' ability to speak actively but a careful observation of whether learners do participate in alternative ways, with a consideration, at the same time, of the interplay among classroom peer relationship, teacher evaluation, and peer evaluation (see Ollin, 2008). From this perspective, Students can actively negotiate multiple classroom roles and identities in the “silent” state (see Morita, 2004) and “actively” participate in the classroom. For example, through the micro analysis of turn taking between teachers and students in Chapter 3, it is found that silence is a kind of implicit interactive resource for participating in the classroom, which can serve as a space for learning, thinking, reflecting, asking for support, expressing understanding difficulties, and cognitive state of educational activities.

Last but not the least, this volume leaves space for an appropriate understanding of the phenomenon of silence and proper measures for effective teaching. The past explicit and active participation showcasing the dominance of Western communicative teaching method may not be the only ideal classroom interaction mode. Silent cognitive participation also has its significance. When classroom activities focus on promoting the deep processing of thinking and reflection, students' silence on the surface, when further studied, actually means more effort of cognitive investment and a complete operation of the internal mechanism, which is beneficial to promoting language learning. For example, Chapter 2 holds that silence is the internal language output of learners and that the construction of tasks should take the silence behavior into account, leaving more space for learners to reflect and to achieve the best state of thinking and speech practice.

To sum up, this volume makes enormous contributions to the study of silence in language education in terms of extending the theoretical horizons, highlighting the importance of the context, and inspiring the classroom teaching practice. In the future, it is recommended that a more systematic investigation of the contextual and psychological factors that affect classroom silence can be conducted from an ecological perspective for silence is situated in different layers of educational ecosystems.

## Author Contributions

PW and HL chose this book together. PW drafted the review. HL provided valuable guidance and made revisions. Both authors contributed to the article and approved the submitted version.

## Conflict of Interest

The authors declare that the research was conducted in the absence of any commercial or financial relationships that could be construed as a potential conflict of interest.
